# Supplementary material for: Short text classification with machine learning in the social sciences: The case of climate change on Twitter
Source: PLoS One. 2023 Sep 29;18(9):e0290762. doi: 10.1371/journal.pone.0290762 (PMC10540966; doi:10.1371/journal.pone.0290762)
Supplement: S1 Appendix — (ZIP) [file pone.0290762.s001.zip › S1_Appendix.pdf]

# SI Appendix: Short Text Classification with Machine Learning in the Social Sciences: The Case of Climate Change on Twitter

## Classification of textual data: The workflow

In the following, we introduce the problem of automated text classification and present the corresponding workflow. We will also discuss various feature extraction methods, as well as text preprocessing techniques.

In the article, we use the following notation. Boldface letters, such as $\boldsymbol{a}$, denote vectors. Capital boldface letters, such as $\boldsymbol{A}$, denote matrices. Meanwhile calligraphic letters, such as $\mathcal{A}$, denote sets. Estimates are denoted as $\hat{a}$. A vector transpose is denoted as $\boldsymbol{a}^T$. Lower indices, such as in $a_j^{(i)}$, are used to denote features, whilst upper indices denote observations. The probability distribution of a variable $a$ is denoted as $p(a)$. Meanwhile, $p(a|b)$ designates the conditional probability distribution of $a$ given $b$ has been observed, where we deliberately do not distinguish between the random variables and their realizations.

The general text classification problem can be formally described as follows. Given a text $\mathcal{T}$ find a label $y$ from a set of available classes $\mathcal{C}$ that best associates with $\mathcal{T}$. Because we do not know the true label $y$, we can only make the best guess $\hat{y}$ which, hopefully, matches $y$. The corresponding workflow is depicted in Fig. 1.

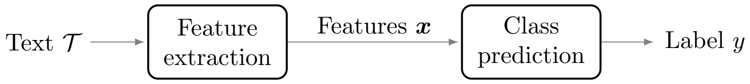


**Fig 1. Text classification workflow.**

In general, a text can refer to a whole document, or to a section, paragraph, or sentence within a document. In this article, we are analysing Twitter data and so we work with tweets. On the Twitter microblogging platform, tweets are the name given to short texts that may contain one or several sentences of anything up to 280 characters (or 140 characters prior to 2012, when for English language the limit was increased due to commonly observed cramming). Generally speaking, tweet texts are considered to be unstructured data. This might be acceptable for a human analyst, who would recognize the context and the meaning in each text and would usually have little or no difficulty with labeling them. However, the unstructured nature of tweets is problematic for an automated text classifier. This requires the conversion of a text into a structured numerical representation. For instance, a lexicon-based classifier needs to tokenize the text, or to break it up into separate words. After the text is tokenized, the classifier can then match the words within it with the key terms from the lexicon. More advanced ML-based classifiers need to map the text into a feature space and create a numerical representation of it, $\boldsymbol{x}$, which is referred to as a *feature vector*. A feature here is referred to as a measurable property of the observed phenomenon, acting as an input to a classifier.

The above process is referred to as *feature extraction* (Fig. 2). The process starts with downloading the tweets which are then parsed for text. This is followed by text

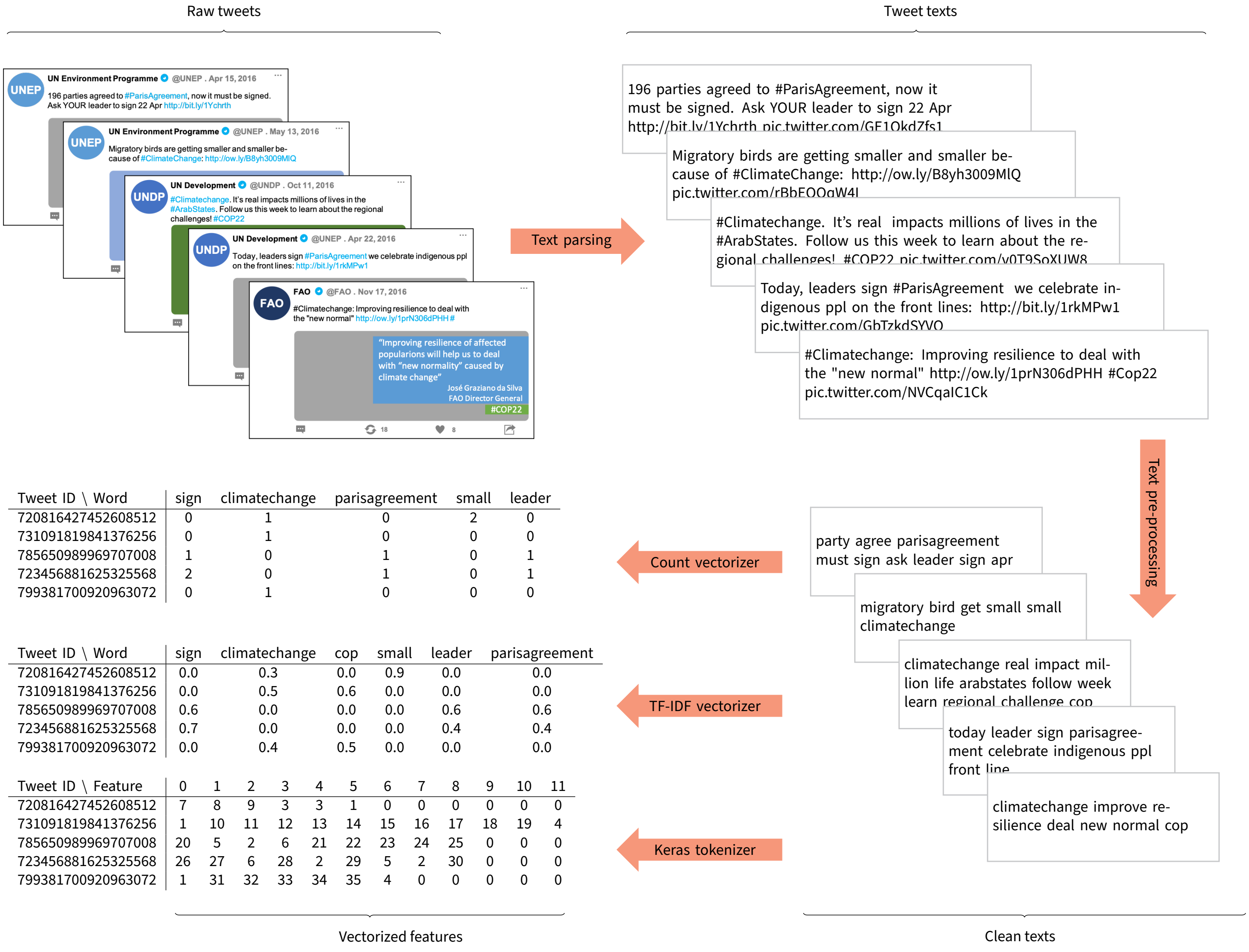


| Tweet ID \ Word | sign | climatechange | parisagreement | small | leader |
|---|---|---|---|---|---|
| 720816427452608512 | 0 | 1 | 0 | 2 | 0 |
| 731091819841376256 | 0 | 1 | 0 | 0 | 0 |
| 785650989969707008 | 1 | 0 | 1 | 0 | 1 |
| 723456881625325568 | 2 | 0 | 1 | 0 | 1 |
| 799381700920963072 | 0 | 1 | 0 | 0 | 0 |

| Tweet ID \ Word | sign | climatechange | cop | small | leader | parisagreement |
|---|---|---|---|---|---|---|
| 720816427452608512 | 0.0 | 0.3 | 0.0 | 0.9 | 0.0 | 0.0 |
| 731091819841376256 | 0.0 | 0.5 | 0.6 | 0.0 | 0.0 | 0.0 |
| 785650989969707008 | 0.6 | 0.0 | 0.0 | 0.0 | 0.6 | 0.6 |
| 723456881625325568 | 0.7 | 0.0 | 0.0 | 0.0 | 0.4 | 0.4 |
| 799381700920963072 | 0.0 | 0.4 | 0.5 | 0.0 | 0.0 | 0.0 |

| Tweet ID \ Feature | 0 | 1 | 2 | 3 | 4 | 5 | 6 | 7 | 8 | 9 | 10 | 11 |
|---|---|---|---|---|---|---|---|---|---|---|---|---|
| 720816427452608512 | 7 | 8 | 9 | 3 | 3 | 1 | 0 | 0 | 0 | 0 | 0 | 0 |
| 731091819841376256 | 1 | 10 | 11 | 12 | 13 | 14 | 15 | 16 | 17 | 18 | 19 | 4 |
| 785650989969707008 | 20 | 5 | 2 | 6 | 21 | 22 | 23 | 24 | 25 | 0 | 0 | 0 |
| 723456881625325568 | 26 | 27 | 6 | 28 | 2 | 29 | 5 | 2 | 30 | 0 | 0 | 0 |
| 799381700920963072 | 1 | 31 | 32 | 33 | 34 | 35 | 4 | 0 | 0 | 0 | 0 | 0 |

**Fig 2. Feature extraction workflow:** from raw tweets to numerical vector representations.

preprocessing [1] of the text extracted from each tweet. Tweets contain considerable noise including redundant content, such as special characters, web links, infrequent terms, and technical words that have been preserved by Twitter. These elements do not contribute to the quality of the text analysis, while increasing its complexity. The text from tweets should therefore be cleaned, making it all lower case, filtering out non-alphabet characters such as hashtag symbols, and removing web links and user names. The punctuation in the tweets also needs to be removed, along with any outstanding single characters and multiple spaces. Short words and stopwords (e.g., such words as ”an” and ”the”) are filtered out, and all the words are further stemmed or lemmatized. Depending on the method of text classification and the type of input it requires, this is then followed by text tokenization and vectorization.

The tokenization and vectorization of texts are typically carried out by means of so-called *bag-of-words* representation. With this approach, all of the input texts are split into separate words. Then, a *count vectorizer* [2] is applied. This vectorizer counts the frequency of appearance of all of the separate words. It therefore forms a global vocabulary of unique words and corresponding per-tweet vectors of indicators of the word presence. These vectors contain the positions of the entries in the global vocabulary. Because of this, they can then be used as input features for a supervised ML model. However, such a (sparse) representation of words might contain lots of noise in terms of frequent common words which provide very little information.

To compensate for this noise in the representation, *TF-IDF weighting* [3] is often used. This technique involves rewarding each word based on its frequency of appearance within a tweet and penalizing it based on an inverse of its frequency across all the tweets in the dataset. In this way, weighting highlights important (frequent) words within the tweet that are also unique within the entire dataset. A computationally efficient implementation of the above two mechanisms, suitable for advanced neural classifiers, is the *Keras tokenizer* [4].

Another alternative representation that is often used is based on *word embeddings* [5, see Chapter 6]. The aforementioned bag-of-words approach creates features that are independent from each other. That is, each new unique word found to occur within the texts adds a new entry to the vocabulary (i.e., the list of words found in all the texts) and therefore adds a new dimension to the latent space. This is problematic for more sophisticated supervised ML methods such as deep neural networks because the more dimensions the feature vector has, the higher the computational complexity of the model. A denser (and fixed-sized) representation is therefore preferable. In this denser representation, the features are embedded into a lower-dimensional space. A denser representation can also capture the context of a word within a sentence and how it is similar to other words. This representation is obtained by multiplying the sparse feature vector with a matrix, where the number of rows corresponds to the desired feature dimensionality. Embedding matrices are obtained by means of a neural network learning from a training set. Nevertheless, we will not investigate the influence of word embeddings here, as this aspect is beyond the scope of the current article.

After the vectorization, we obtain a dataset in a matrix format: columns represent features, with the last column designating the label, while rows represent observations,

$$\mathcal{D} = \{(\boldsymbol{x}^{(i)}, y^{(i)})\}_{i=1}^{N}, \tag{1}$$

where $i$ is an observation index that corresponds to a single tweet from the $N$ tweets in the dataset. Each observation is a tuple $(\boldsymbol{x}^{(i)}, y^{(i)})$, where $\boldsymbol{x}^{(i)}$ is a feature vector consisting of $M$ features and $y^{(i)}$ is a (scalar) label (see Fig. 3 for an illustration). Without loss of generality, we consider the labels to be binary variables, coded as “1” if a given tweet is about climate change, and “0” otherwise. Generalization to multiple

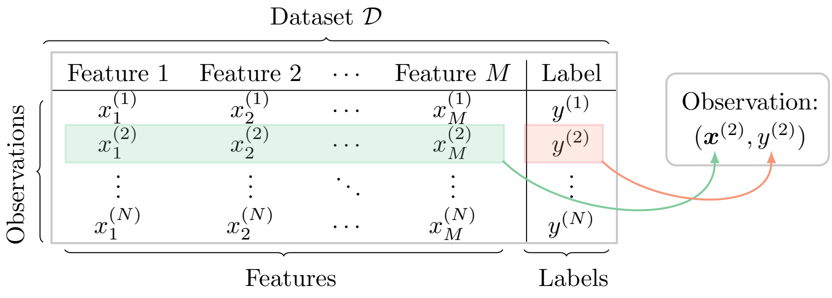


**Fig 3. Dataset format:** a set of observations, each consisting of a feature vector and a label.

classes can be done by means of the so-called *one-hot* encoding, transforming the problem into binary classification for each class vs. all the other classes.

Formally, the classification task is defined as learning an unknown *predictor function* $f(\cdot)$, that maps a feature vector $\boldsymbol{x}$ into a predicted label $y$ for any possible observation $(\boldsymbol{x}, y)$. However, there may not be a perfect mapping between the features and the labels. This is especially considering the fact that the features are chosen by an analyst and are therefore subject to omitted variables. The latter results in variations in the data due to these "unmodeled" variables which manifest themselves as seemingly random noise. In practice, therefore, a *good* mapping $\hat{f}(\cdot)$ (or one that approximates the true predictor function $f(\cdot)$)—referred to as a model or a hypothesis—is obtained by means of an automated classifier instead. The classifier then outputs a predicted label $\hat{y} = \hat{f}(\boldsymbol{x}^{(i)})$ which should potentially match the true label $y$ most of the time.

# Illustrations of deep learning

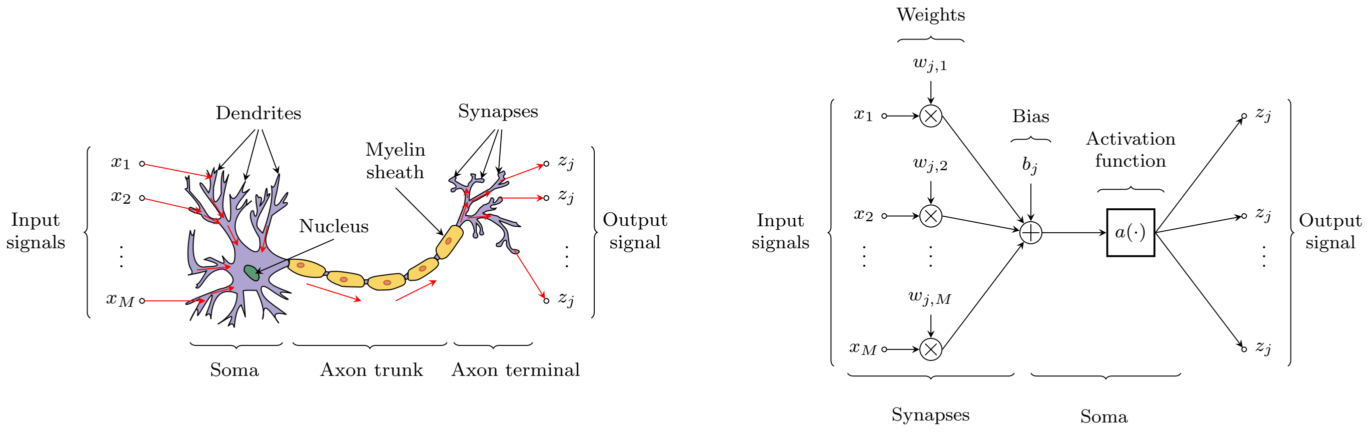


**(a)** Biological neuron.

**(b)** Artificial neuron.

**Fig 4. Modeling of a neuron.** A biological neuron (a) processing an aggregation of its input signals is modeled as an artificial neuron (b) weighing its inputs, adding a bias and applying a non-linear activation function.

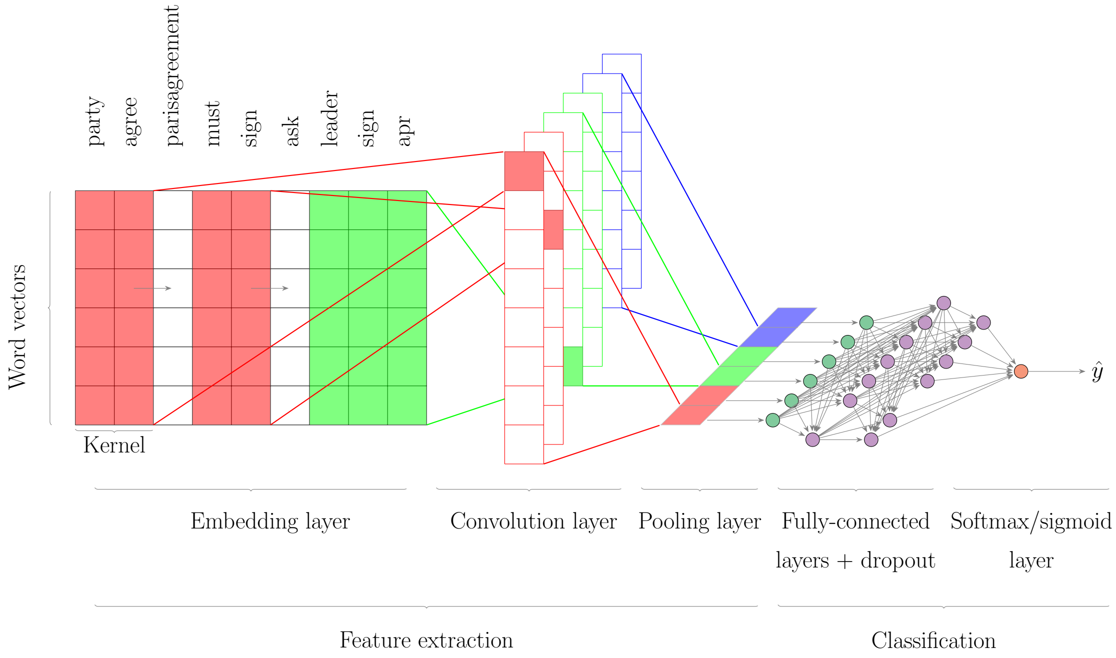


**Fig 5. An illustrative architecture of a CNN.** A kernel is convolved over the output of the embedding layer to produce feature maps. These are convolved by a kernel and pooled to provide features for the subsequent classification by a fully-connected layer.

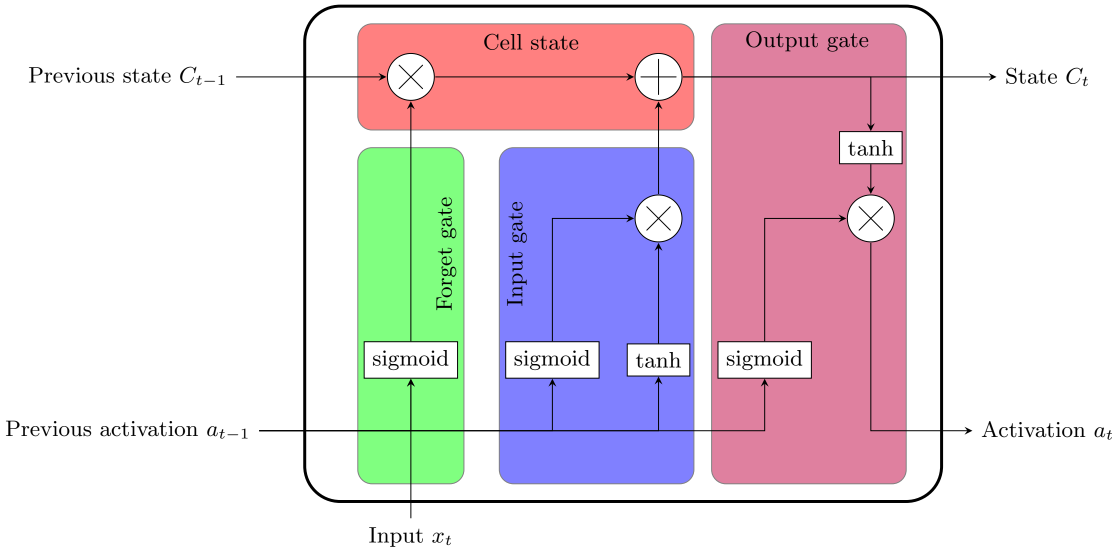


**Fig 6. Architecture of an LSTM neural cell.** Its state is represented by a horizontal line running from time instance $t-1$ to time instance $t$. The cell input is concatenated with the previous instance's activation and goes through the cell's gates to result in the output cell state and activation function at the current instance.

## Statistical tests

In this section, we present the statistical validation of the performance results of the classification methods under consideration. To that end, we investigate whether the

| | | Model 2 | |
|---|---|---|---|
| | | Classified incorrectly | Classified correctly |
| Model 1 | Classified incorrectly | Both incorrect ($N_{0,0}$) | Model 1 incorrect ($N_{0,1}$) |
| | Classified correctly | Model 2 incorrect ($N_{1,0}$) | Both correct ($N_{1,1}$) |

**Fig 7. Contingency table containing counts of four sets of test observations.** The categorization of each observation is based on the comparison of the label predicted by the two models with the true one.

differences in performance are statistically significant by relying on the widely-used non-parametric McNemar test [6]. This is done by conducting the pair-wise comparison of the classifiers in terms of the contingency table [7] which assesses their performance vs. the true labels on the test set. The contingency table is illustrated in Fig. 7, showing that the observations in the test set fall into one of four sets: misclassified by both models (with $N_{0,0}$ entries), misclassified by model 1, but not model 2 (with $N_{0,1}$ entries), misclassified by model 2, but not model 1 (with $N_{1,0}$ entries), classified correctly by both models (with $N_{1,1}$ entries). Based on this, McNemar's test checks for the null hypothesis of the marginal homogeneity of the outcomes, viz., $N_{0,1} = N_{1,0}$. To verify the letter, for each pair of classifiers, we compute the McNemar statistic

$$\mu = \frac{(N_{0,1} - N_{1,0})^2}{N_{0,1} + N_{1,0}}. \quad (2)$$

Given a sufficiently large number of discordants in the test set (i.e., observations falling into $N_{0,1}$ and $N_{1,0}$), this statistic follows the $\chi^2$ distribution with a single degree of freedom. If $\mu$ is statistically significant, we have grounds to reject the null hypothesis and conclude that the marginal proportions in the contingency table are statistically different, viz. the two classifiers have different error rates. Otherwise, we conclude that the null hypothesis cannot be rejected and the two models have very similar performance.

The results of McNemar's test on the test set withheld from the balanced dataset are presented in Fig. 8. Namely, Fig 8a shows the McNemar test statistic of the pair-wise comparison of the model performance. Meanwhile, Fig. 8b shows the corresponding p-values. Setting the significance value to $p = 0.05$, we get from the $\chi^2$ distribution density the corresponding threshold on the test statistic of $\mu^* = 3.841$. Using these values, we can make a decision whether to reject or accept the null hypothesis in every pair-wise performance comparison. The results of such comparison are presented in Fig. 8c.

It can be clearly seen from the figure that for most classifier pairs we cannot reject the null hypothesis, meaning that the difference in their performance is not statistically significant. There are two exceptions—lexicon-based classifier and KNN—whose performance significantly differs from the rest of the models. This is in line with our observations from Table 4 in the manuscript which clearly shows that lexicon and KNN are performance outliers, exhibiting the lowest F1 score among the tested models. These observations underpin our conclusion that the more balanced the data, the better is the performance of the classifiers, regardless of whether those are representatives of traditional machine learning or deep learning. The difference in their performance becomes negligible, with the exception of the two aforementioned less capable classifiers.

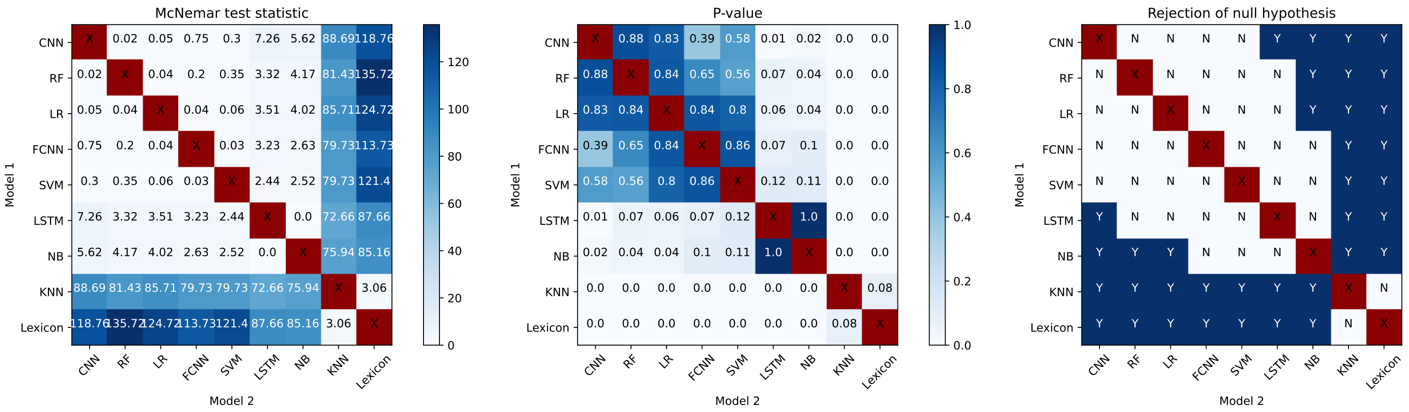


**(a)** McNemar test statistics. **(b)** P-values. **(c)** Null hypothesis rejection decisions.

**Fig 8. Results of McNemar's test.** Pair-wise test statistics (a) and corresponding p-values (b) provide a basis for a decision on the rejection of the null hypothesis of statistically similar performance of the classifiers under consideration (c).

# References


1. Denny MJ, Spirling A. Text preprocessing for unsupervised learning: Why it matters, when it misleads, and what to do about it. Political Analysis. 2018;26(2):168–189.

2. du Boisberranger J, den Bossche JV, Estève L, Fan TJ, Gramfort A, Grisel O, et al.. Count Vectorizer; 2023. `https://scikit-learn.org/stable/modules/generated/sklearn.feature_extraction.text.CountVectorizer.html`, accessed Apr. 2023.

3. Salton G, Buckley C. Term-weighting approaches in automatic text retrieval. Information processing & management. 1988;24(5):513–523.

4. Chollet F, Görner M, Gulli A, Moolayil J, Heaton J, Allaire JJ, et al.. TensorFlow Tokenizer; 2023. `https://www.tensorflow.org/api_docs/python/tf/keras/preprocessing/text/Tokenizer`, accessed Apr. 2023.

5. Jurafsky D. Speech & language processing. Pearson Education India; 2000.

6. McNemar Q. Note on the sampling error of the difference between correlated proportions or percentages. Psychometrika. 1947;12(2):153–157.

7. Pearson K. On the theory of contingency and its relation to association and normal correlation. In: Drapers' Company research memoirs. Biometric series. vol. 1. Dalau and Co.; 1904.
