# Supplementary material for: Short text classification with machine learning in the social sciences: The case of climate change on Twitter
Source: PLoS One. 2023 Sep 29;18(9):e0290762. doi: 10.1371/journal.pone.0290762 (PMC10540966; doi:10.1371/journal.pone.0290762)
Supplement: Attachment — Submitted filename: plos_one_short_text_classification_rebuttal.pdf [file pone.0290762.s002.pdf]

PONE-D-22-35683
Short Text Classification with Machine Learning in the Social Sciences: The Case of Climate Change on Twitter

March 22, 2023

Response to the Editor and Reviewers

We thank the Editor and Reviewers for providing constructive feedback and excellent suggestions on how to further improve the article. We carefully considered the feedback when reworking the paper. We hope that the changes we have made are up to the reviewers' satisfaction. In the following, we present our responses point-by-point. The changes we made in the manuscript are highlighted in blue. In our letter, the eventual numbers in references, equations, figures and tables refer to the ones in the new version of the manuscript (unless stated otherwise).

Editor

Editor Point E 1 — Please conduct statistical tests, as this is the only way to prove that results generated by one method are significantly better than the ones obtained by other approaches included in comparative analysis.

Reply: We thank the editor for the useful suggestion. To address it, we have conducted pair-wise McNemar tests for each pair of the classifiers. In this way, we have obtained a table assessing which of the differences in performance between the classifiers are statistically significant. We now discuss the results of the tests in the manuscript and have added the results of the test to the SI appendix, which is referenced in the main manuscript. See a more detailed elaboration in the response to reviewer point R 3.5.

Editor Point E 2 — The GITHUB link with dataset and source code that you provided is broken. Please, make sure that you share all relevant details, including the dataset and the source code, so that other researchers' could try to even further improve achieved results.

Reply: We thank the editor for pointing us to this issue. In the manuscript, we have now added both a link to the dataset and a link to the code to ensure the replicability of the results. In footnote 6, we have added the following:

For the sake of reproducibility, the notebooks containing the codes are available at our repository `https://github.com/shikarina/short_text_classification`.

Meanwhile, in footnote 7, we have added the following statement:
The dataset is available at https://zenodo.org/record/7633599#.Y-lbTS8w1qs.

Reviewer 1

Reviewer Point R 1.1 — Avoid using we/our throughout the manuscript.

Reply: We are grateful to Reviewer 1 for bringing this to our attention. We understand that Reviewer 1 might be coming from a discipline where personal pronouns are not used in academic publications. In social sciences, however, this is very common, and vice versa it rather uncommon to write "this article argues/suggests..." This is the feedback which we also received when the article was proofread by a native speaker. Therefore, we would prefer to keep the personal pronouns throughout. Of course, if the Editor thinks that we should remove them, we will do that in the next iteration.

Reviewer Point R 1.2 — Literature review should be expanded with recent machine learning models tuned by metaheuristics optimizer, that were applied to the text classification problem. Include the following references:

- https://www.mdpi.com/2227-7390/9/16/1929
- https://link.springer.com/chapter/10.1007/978-981-19-3035-5_56
- https://www.tandfonline.com/doi/full/10.1080/08839514.2021.2004345
- https://www.mdpi.com/2227-7390/10/22/4173
- https://www.inderscienceonline.com/doi/abs/10.1504/IJCAT.2022.123237.

Reply: We thank the reviewer for these suggestions and for bringing our attention to the issue of hyperparameter tuning. Indeed, there are more methods for hyperparameter tuning in machine learning than Bayesian optimization, including grid search and metaheuristics of various kinds. To address this very useful comment, we have added a short discussion about that in footnote 10 (the Performance Assessment section of the manuscript), where we have cited, i.a., some of the research referenced above.

Reviewer Point R 1.3 — Line 39, unsupervised, line 44, supervised, and line 47, deep learning - is it necessary to mark them in italic font?

Reply: We thank the reviewer for pointing this out. The objective of a different font was to emphasise certain concepts. We agree that there were too many words in italic. We remove most of the italic font using it only for the keywords that we want our readers to remember when reading the article.

Reviewer Point R 1.4 — Add the structure of the paper at the end of the introduction section.

Reply: We agree with this suggestion. In response, we have added such a paragraph at the end of the introductory section.

Reviewer Point R 1.5 — Make sure that each parameter in every equation has been explained in the text.

Reply: We thank the reviewer for this suggestion. We have now gone through all the equations in the manuscript and made sure that all the parameters are explained in the text. The corresponding changes are highlighted in blue color throughout the descriptions of equations in the text.

Reviewer Point R 1.6 — Line 155 - again, is it necessary to use italic font? Also consider is it required to use it in other places throughout the text.

Reply: We thank the reviewer for pointing this out. We remove the excessive italic font.

Reviewer Point R 1.7 — Figure 1 is not adding anything valuable to the manuscript, and should be removed.

Reply: We agree with the reviewer on this and have now removed Figure 1 from the paper, as requested.

Reviewer Point R 1.8 — In my opinion, tables with the results should not be sorted by the preferred metric. Rather, highlight the best result in each category in bold. For example, in Table 2, LR F1 score, KNN precision, SVM recall etc.

Reply: We thank Reviewer 1 for this suggestion. However, we would like to point out that we consider F1 score as the main performance metric (as motivated in the manuscript). Therefore, for us the best-performing classifier is determined by its F1 performance. The rest of the performance metrics are added as additional information for an advanced reader. For that reason, we prefer to keep the table formats unchanged, highlighting in bold only the preferred performance metric.

Reviewer Point R 1.9 — Discuss the limitations of each observed classifier.

Reply: This is an excellent suggestion, which we have taken on board. We have added a paragraph discussing the advantages and disadvantages of each classifier in the respective subsections in the manuscript. The corresponding changes are highlighted in blue color throughout the descriptions of the classifiers.

Reviewer 2

Reviewer Point R 2.1 — The article deals with text classification using ML models. The main contribution of the paper is a comparison between various ML models. The authors are reviewing different automated text classification methods. They use different optimizers for hyperparameter tuning. Having that in mind, authors should mention (or even consider using in the future work) recent articles which use ML models and metaheuristics (for parameter tuning and feature selection) in language processing, for example:

- Bacanin, N.; Zivkovic, M.; Stoean, C.; Antonijevic, M.; Janicijevic, S.; Sarac, M.; Strumberger, I. Application of Natural Language Processing and Machine Learning Boosted with Swarm Intelligence for Spam Email Filtering. Mathematics 2022, 10, 4173. https://doi.org/10.3390/math10224173
- Nandanwar, A.K.; Choudhary, J. Semantic Features with Contextual Knowledge-Based Web Page Categorization Using the GloVe Model and Stacked BiLSTM. Symmetry (Basel). 2021, 13, 1772
- El-Alami, F. zahra; Ouatik El Alaoui, S.; En Nahnahi, N. Contextual Semantic Embeddings Based on Fine-Tuned AraBERT Model for Arabic Text Multi-Class Categorization. J. King Saud Univ. - Comput. Inf. Sci. 2022, 34, 8422–8428

Reply: We appreciate these suggestions and agree that metaheuristics for parameter tuning and feature selection are a prominent direction in the field of machine learning. We will for sure consider this for our future work. Meanwhile, some of the papers the reviewer suggests deal with word embedding models which are considered (and mentioned) as future work in our manuscript. Therefore, to accommodate the reviewer's suggestion, we have cited some of the proposed papers in footnote 10 and on page 16:

Note that there are other known optimization algorithms used for hyperparameter tuning, e.g., random search (66) or metaheuristics, such as genetic algorithm (67), simulated annealing (68), differential evolution (69) and swarm intelligence (70).

We have also mentioned the topic of hyperparameter tuning as a future research avenue in the Conclusions section of the manuscript.

Reviewer 3

Reviewer Point R 3.1 — The paper puts forward a data set with tweets from 8 UN organizations. The statements are manually labelled as being or not related to climate change. The data set itself represents an important advancement not only for replicating the current experiment, but even for being used as benchmark in the future. However, the link provided for the data set is not valid. The authors are advised to use a platform like figshare.com for providing the data (I underline that the current web link is not even valid).

Reply: We thank Reviewer 3 for pointing out these issues. We agree with the reviewer that our dataset itself represents an important advancement, useful for benchmarking in the future. We did not disclose the link to the dataset and codes in the previous version of the manuscript to not make them prematurely available to the public. In addition, a major obstacle to sharing the dataset was the difficulty with obtaining a permission for publishing Twitter data, as terms and conditions postulated by Twitter for the use of its Academic API prohibit the direct publishing of tweets. Another aspect to address was the potential presence of private/sensitive information in tweets, publishing which might have led to the identifiability of physical persons, which would have violated the GDPR and ethical guidelines. For more information, see `datajournalism.com/read/longreads/data-journalism-and-the-ethics-of-publishing-twitter-data`.

Nevertheless, Reviewer 3's request has inspired us to look into this issue in more depth. It seems that the current best practice is to publish Twitter data in the *dehydrated* form (i.e., only publishing tweet IDs, not text or other meta-data), which will be used by researchers who will

need to *hydrate* it (i.e., download the specific tweets by their IDs using, e.g., the Hydrator app - `https://github.com/DocNow/hydrator/releases`).

In response the reviewer's important request, we have thus published our data in the dehydrated form. Our full and labeled datasets are now available at the Open Science repository. We have also added the corresponding link to footnote 7 in the Results section of the manuscript:

The dataset is available at `https://zenodo.org/record/7633599#.Y-lbTS8w1qs`.

Reviewer Point R 3.2 — The experiments are nicely described. Particularly, the experiment with the controlled balance ratio was very interesting.

Reply: We appreciate Reviewer 3's endorsement of our Results section. We feel encouraged and hope that the rest of the reviewer's comments are also addressed to her/his satisfaction.

Reviewer Point R 3.3 — Please clarify whether the same balance between the classes that exists in the entire data set is also kept in all data sets, training, validation and test.

Reply: We thank the reviewer for this question. Indeed, the same balance ratio is kept for the three parts of the dataset. We have now clarified this in footnote 12 in the Results section:

Note that for each balance-ratio point in this experiment, the balance between the classes in the training, validation and test sets is kept the same.

Reviewer Point R 3.4 — Please also provide more information on the parameters that were used within the parameter tuning for each model in turn. The conclusion regarding the better and worse models is quite strong and it may be the case that the parameter tuning might be more advantageous for some models than for others.

Reply: This is a very good point. To begin with, regarding the parameters used for the hyperparameter tuning, we would like to point out that the parameters we performed tuning over are presented in the last paragraph of each section that discusses a particular classification method. For example, for the random forest classifier we wrote "*For our empirical analysis, we adjusted the maximum depth of the decision trees and the number of estimators in order to optimize the RF classifier's performance*", which captures the available parameters in the method's SciKit-Learn implementation which we swept to optimize the classifier's performance. Similar descriptions are added in other subsections describing other classifiers.

Meanwhile, we agree with the reviewer that the benefit from the hyperparameter tuning might differ from one classifier to another. Since the training of a complex classifier is often a non-convex optimization problem, the "tuned" parameters might in fact be local optima and hence might not guarantee the best possible performance. Thus, it might not be even possible to compare the classifiers in a completely fair way – various classifiers will always be sub-optimal to a different extent. Moreover, the complexity and time of the tuning should be considered. Hence, when taking this into account, it might make more sense to get a decent classifier in a reasonable tuning time, rather than a close-to-perfect classifier whose tuning takes an extremely long time.

Nevertheless, we would like to point out that in our work we are using state-of-the-art Bayesian optimization techniques that have proven their robustness and efficiency in literature. See, e.g., the references below, as well as many more other works on hyperparameter tuning.

- Snoek, Jasper, Hugo Larochelle, and Ryan P. Adams. "Practical Bayesian optimization of machine learning algorithms." Advances in neural information processing systems 25 (2012).

- Chai, Chen, et al. "An automated machine learning (AutoML) method for driving distraction detection based on lane-keeping performance." arXiv preprint arXiv:2103.08311 (2021).
- Witt, Nils, and Christin Seifert. "Understanding the influence of hyperparameters on text embeddings for text classification tasks." Research and Advanced Technology for Digital Libraries: 21st International Conference on Theory and Practice of Digital Libraries, TPDL 2017, Thessaloniki, Greece, September 18-21, 2017.

Therefore, given all the above, we believe that the tuning of our classifiers is conducted to a reasonable extent and should suffice to make an appropriate comparison. However, to acknowledge the reviewer's point, we have mentioned the issue of hyperparameter tuning in the future research part of the concluding section. We agree that more research is needed on the performance of hyperparameter tuning in different algorithms.

Reviewer Point R 3.5 — Some statistical tests could highlight whether the differences in results are significant or not, be that there are enough results for such tests.

Reply: We are grateful to Reviewer 3 for this useful suggestion, as it points at an important aspect which deserves to be added to this work. To address the reviewer's concern about the statistical significance of the difference in performance of the considered classifiers, we have performed pair-wise McNemar's statistical test for each pair of these algorithms on the balanced test set. We have added a corresponding section in the SI appendix (p. 6), where we describe the methodology and the results of the test.

Our results suggest that the difference in the performance of most of the methods is not statistically significant, except for the two less capable classifiers: KNN and lexicon. For the best 5-7 methods, the error rates are in the same proportions, hence the performance is similar. This complements and underlines our conclusion that the performance difference is small for the balanced dataset, and that it thus appears warranted to use traditional machine-learning methods instead of computationally intense deep learning.

Aside from the new section in the SI appendix, we have added the following paragraph in the Results section in the manuscript (p. 19):

It is however unclear whether the differences in the performance of these classifiers is statistically significant or not. To investigate this, we conduct pair-wise McNemar's tests (82) on every possible pair of classifiers under consideration. The corresponding methodology is described in the SI Appendix, where Fig 8 presents the results of the statistical tests. The results show that the performance of almost all the considered methods does not significantly differ from one another, with the exception of KNN and lexicon classifiers. That is, for scenarios where the dataset is balanced, it matters less which classification algorithm is chosen (with the exception of the KNN and lexicon classifiers).

Admin

Admin Point A 1 — Please ensure that your manuscript meets PLOS ONE's style requirements, including those for file naming.

Reply: We edited formatted to meet PLOS ONE's style requirements.

Admin Point A 2 — Thank you for stating the following financial disclosure: "This work was supported by the project: "Glocalizing Climate Governance: The role of Integrated Governance for a Just and Legitimate Adaptation to Climate Risks (GlocalClim)" funded by Formas under grant number 2018-01705. Svenska Forskningsrådet Formas, Grant/Award Number: 2018-01705."

Please state what role the funders took in the study. If the funders had no role, please state: "The funders had no role in study design, data collection and analysis, decision to publish, or preparation of the manuscript." If this statement is not correct you must amend it as needed.

Please include this amended Role of Funder statement in your cover letter; we will change the online submission form on your behalf.

Reply: We added the corresponding changes. Now the financial disclosure states:

This work was supported by the project: "Glocalizing Climate Governance: The role of Integrated Governance for a Just and Legitimate Adaptation to Climate Risks (GlocalClim)" funded by Formas under grant number 2018-01705 (Svenska Forskningsrådet Formas, Grant/Award Number: 2018-01705). The funder had no role in study design, data collection and analysis, decision to publish, or preparation of the manuscript.

Admin Point A 3 — In your Data Availability statement, you have not specified where the minimal data set underlying the results described in your manuscript can be found. PLOS defines a study's minimal data set as the underlying data used to reach the conclusions drawn in the manuscript and any additional data required to replicate the reported study findings in their entirety. All PLOS journals require that the minimal data set be made fully available. For more information about our data policy, please see `http://journals.plos.org/plosone/s/data-availability`.

Upon re-submitting your revised manuscript, please upload your study's minimal underlying data set as either Supporting Information files or to a stable, public repository and include the relevant URLs, DOIs, or accession numbers within your revised cover letter. For a list of acceptable repositories, please see `http://journals.plos.org/plosone/s/data-availability#loc-recommended-repositories`. Any potentially identifying patient information must be fully anonymized.

Important: If there are ethical or legal restrictions to sharing your data publicly, please explain these restrictions in detail. Please see our guidelines for more information on what we consider unacceptable restrictions to publicly sharing data:

`http://journals.plos.org/plosone/s/data-availability#loc-unacceptable-data-access-restrictio`
Note that it is not acceptable for the authors to be the sole named individuals responsible for ensuring data access.

We will update your Data Availability statement to reflect the information you provide in your cover letter.

Reply: We have now made this dataset available, as described above. We have saved a dehydrated dataset online and provided a link to it. We did not provide the original data in order to comply with Twitter API terms and conditions, which strictly forbid users to publish Twitter data in the original form. The Data Availability statement is now complemented with the following:

The dataset is available at `https://zenodo.org/record/7633599#.Y-lbTS8w1qs`.

Admin Point A 4 — We note that Supporting Figure 1 in your submission contain copyrighted images. All PLOS content is published under the Creative Commons Attribution License (CC BY 4.0), which means that the manuscript, images, and Supporting Information files will be freely available online,

and any third party is permitted to access, download, copy, distribute, and use these materials in any way, even commercially, with proper attribution. For more information, see our copyright guidelines: http://journals.plos.org/plosone/s/licenses-and-copyright.

We require you to either (1) present written permission from the copyright holder to publish these figures specifically under the CC BY 4.0 license, or (2) remove the figures from your submission:

(1) You may seek permission from the original copyright holder of Supporting Figure 1 to publish the content specifically under the CC BY 4.0 license.

We recommend that you contact the original copyright holder with the Content Permission Form (http://journals.plos.org/plosone/s/file?id=7c09/content-permission-form.pdf) and the following text:

"I request permission for the open-access journal PLOS ONE to publish XXX under the Creative Commons Attribution License (CCAL) CC BY 4.0 (http://creativecommons.org/licenses/by/4.0/). Please be aware that this license allows unrestricted use and distribution, even commercially, by third parties. Please reply and provide explicit written permission to publish XXX under a CC BY license and complete the attached form."

Please upload the completed Content Permission Form or other proof of granted permissions as an ""Other"" file with your submission.

In the figure caption of the copyrighted figure, please include the following text: "Reprinted from [ref] under a CC BY license, with permission from [name of publisher], original copyright [original copyright year]."

(2) If you are unable to obtain permission from the original copyright holder to publish these figures under the CC BY 4.0 license or if the copyright holder's requirements are incompatible with the CC BY 4.0 license, please either i) remove the figure or ii) supply a replacement figure that complies with the CC BY 4.0 license. Please check copyright information on all replacement figures and update the figure caption with source information. If applicable, please specify in the figure caption text when a figure is similar but not identical to the original image and is therefore for illustrative purposes only.

Reply: Fig. 1 in the SI appendix does not have any copyrighted images. It is a scheme showing the text classification workflow. Might it be the case that what was meant here is Fig. 2 in the SI appendix, where there are screenshots from Twitter? If that is the case, we would like to point out that tweets per se are very short, and hence very hard to become copyrightable. For more details, see here:

https://www.wipo.int/wipo_magazine/en/2009/04/article_0005.html

If the problem is the actual image in the tweet by FAO, shown in the figure as an input to our feature extraction workflow, then to address this we have modified this potentially copyrighted image. It has now become significantly different from that in the original tweet by FAO (see below). We hope this resolves the issue.

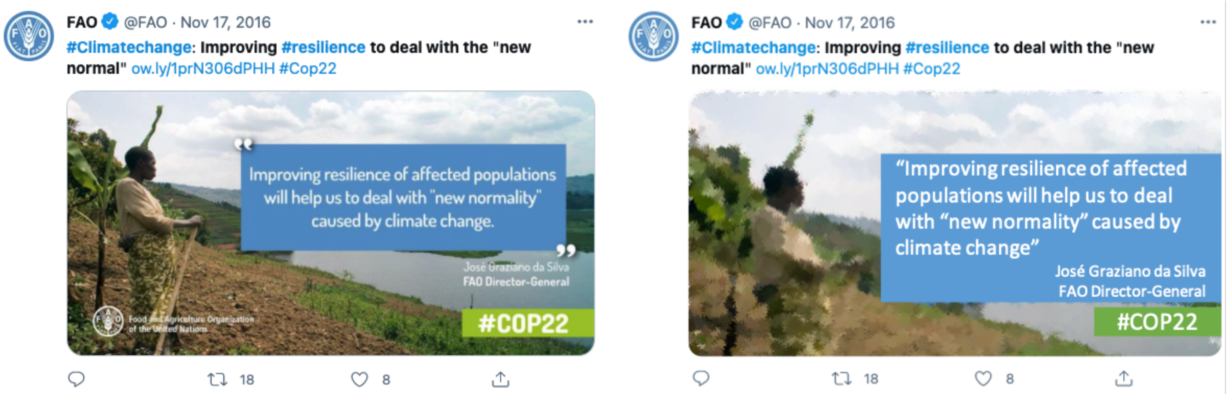

(a) Original tweet by FAO.

(b) Modified version for the SI appendix.

Figure 1: Modification of the potentially copyrightable image in the FAO tweet.
